# Hidden Nickel Deficiency? Nickel Fertilization via Soil Improves Nitrogen Metabolism and Grain Yield in Soybean Genotypes

**DOI:** 10.3389/fpls.2018.00614

**Published:** 2018-05-08

**Authors:** Douglas Siqueira Freitas, Bruna Wurr Rodak, André Rodrigues dos Reis, Fabio de Barros Reis, Teotonio Soares de Carvalho, Joachim Schulze, Marco A. Carbone Carneiro, Luiz R. Guimarães Guilherme

**Affiliations:** ^1^Laboratory of Soil Microbiology and Environmental Geochemistry, Department of Soil Science, Federal University of Lavras, Lavras, Brazil; ^2^Laboratory of Biology, School of Science and Engineering, São Paulo State University, Tupã, Brazil; ^3^CropSolutions Agricultural Research Center, São Gabriel do Oeste, Brazil; ^4^Laboratory of Plant Nutrition and Crop Physiology, Department of Crop Science, Faculty of Agriculture, University of Göttingen, Göttingen, Germany

**Keywords:** ammonia, biological nitrogen fixation, *Glycine max*, photosynthesis, urea, urease activity, ureides

## Abstract

Nickel (Ni)—a component of urease and hydrogenase—was the latest nutrient to be recognized as an essential element for plants. However, to date there are no records of Ni deficiency for annual species cultivated under field conditions, possibly because of the non-appearance of obvious and distinctive symptoms, i.e., a hidden (or latent) deficiency. Soybean, a crop cultivated on soils poor in extractable Ni, has a high dependence on biological nitrogen fixation (BNF), in which Ni plays a key role. Thus, we hypothesized that Ni fertilization in soybean genotypes results in a better nitrogen physiological function and in higher grain production due to the hidden deficiency of this micronutrient. To verify this hypothesis, two simultaneous experiments were carried out, under greenhouse and field conditions, with Ni supply of 0.0 or 0.5 mg of Ni kg^−1^ of soil. For this, we used 15 soybean genotypes and two soybean isogenic lines (urease positive, *Eu3*; urease activity-null, *eu3-a*, formerly *eu3-e1*). Plants were evaluated for yield, Ni and N concentration, photosynthesis, and N metabolism. Nickel fertilization resulted in greater grain yield in some genotypes, indicating the hidden deficiency of Ni in both conditions. Yield gains of up to 2.9 g per plant in greenhouse and up to 1,502 kg ha^−1^ in field conditions were associated with a promoted N metabolism, namely, leaf N concentration, ammonia, ureides, urea, and urease activity, which separated the genotypes into groups of Ni responsiveness. Nickel supply also positively affected photosynthesis in the genotypes, never causing detrimental effects, except for the *eu3-a* mutant, which due to the absence of ureolytic activity accumulated excess urea in leaves and had reduced yield. In summary, the effect of Ni on the plants was positive and the extent of this effect was controlled by genotype-environment interaction. The application of 0.5 mg kg^−1^ of Ni resulted in safe levels of this element in grains for human health consumption. Including Ni applications in fertilization programs may provide significant yield benefits in soybean production on low Ni soil. This might also be the case for other annual crops, especially legumes.

## Introduction

Nickel (Ni) was the latest element to be included in the list of essential nutrients to plants. The first evidence of its essentiality was verified in soybean plants (*Glycine max* [L.] Merrill) in 1983, under controlled conditions of Ni depletion, when these plants accumulated toxic concentrations of urea in leaflet tips (Eskew et al., 1983). The evidence that Ni is an essential plant micronutrient was confirmed four years later, when after three successive generations of growing barley plants (*Hordeum vulgare* L.) in Ni-depleted controlled conditions, these plants failed to produce viable grains (Brown et al., 1987).

In plants, Ni constitutes the active site of two metalloenzymes that are directly involved in nitrogen metabolism (N metabolism): urease (Dixon et al., 1975) and hydrogenase (Evans et al., 1987). Urease is responsible for hydrolysis of urea into two molecules of ammonia and one of carbon dioxide (Witte, 2011; Polacco et al., 2013), while legume plants in symbiosis with N_2_-fixing bacteria form root nodules, in which hydrogenase catalyzes the oxidation of molecular hydrogen (H_2_) into protons and electrons (Shafaat et al., 2013; Bagyinka, 2014; Brazzolotto et al., 2016).

For legume plants that are highly efficient in biological nitrogen fixation (BNF), such as soybean, urease and hydrogenase have a very significant role. When nitrogenase reduces atmospheric N_2_, these Ni metalloenzymes acts in two downstream biological reactions. Most N fixed in root nodules, as ammonia, is converted into ureides (allantoin and allantoic acid), which are the main forms of N exported to aboveground plant parts (Collier and Tegeder, 2012). Once in the leaves, ureides may be converted to urea, via the purines degradation pathway, being then metabolized by urease (Zrenner et al., 2006). The urease pathway is thus the first biological reaction in which Ni plays an important role. Nitrogenase not only reduces N_2_ to ammonia, but also produces molecular hydrogen. The produced hydrogen gas is re-oxidized by the hydrogenase enzyme, recovering a certain amount of the energy used for the previous reduction by nitrogenase (González-Guerrero et al., 2014). The hydrogenase pathway is the second biological reaction in which Ni is required.

The importance of Ni as a micronutrient has been demonstrated under greenhouse conditions (Dixon et al., 1975; Eskew et al., 1983; Brown et al., 1987; Evans et al., 1987). Subsequently, it was possible to attribute the “mouse-ear” symptomology on pecan orchards (*Carya illinoinensis* [Wangenh.] K. Koch) to Ni deficiency (Wood et al., 2004a,b, 2006). Ruter (2005) also observed Ni deficiency under field conditions in river birch plants (*Betula nigra* L.).

Nickel deficiency in these plants occurred in soils poor in extractable Ni. Even though plants usually have a low demand for this micronutrient (Seregin and Kozhevnikova, 2006), it can be expected that Ni-poor soils might also cause a hidden (or latent) deficiency in other plant species (Wood, 2013). Under such circumstances, plants would not express their maximum growth potential even without any deficiency symptoms, as visible lesions are the last step of a series of metabolic problems.

Soybean is a summer crop of a great economic and social importance worldwide, being the major source of vegetable oil (Food Agriculture Organization of the United Nations, 2017). Cultivation of this crop is common on soils low in extractable Ni (Licht et al., 2006; Roca et al., 2008; Morrison et al., 2009; Jaworska et al., 2013; Dabkowska-Naskret et al., 2014; Rodak et al., 2015). Because of that, a hidden deficiency of this micronutrient can be predicted. In addition, the high dependence of this legume on BNF may further increase its demand for Ni.

Recent studies have demonstrated that fertilization with Ni can increase N assimilation and N metabolite levels in plants (Tan et al., 2000; Khoshgoftarmanesh et al., 2011; Hosseini and Khoshgoftarmanesh, 2013; Dalir and Khoshgoftarmanesh, 2015; Uruç Parlak, 2016). In soybean, this effect in N metabolism (Kutman et al., 2013, 2014) as well as in BNF stimulation (González-Guerrero et al., 2014; Lavres et al., 2016; Macedo et al., 2016) is also observed, yet these results were obtained under artificial growth conditions (greenhouse with soil or nutrient solution). Furthermore, only a limited number of genotypes were tested. Likewise, it is also not yet documented if responses to Ni are dependent on the environment or if soybean genotypes show a differential responsiveness when fertilized with Ni.

Considering the dependence of soybean on BNF and an often-low content of extractable Ni in soils, the hypothesis of this study was that Ni fertilization in soybean genotypes, under greenhouse and field conditions, promotes both growth and physiological activity, alleviating situations of hidden Ni deficiency.

## Materials and methods

### Experimental design

In order to verify Ni-fertilization effects in soybean plants, two simultaneous experiments were performed (from November 2015 to March 2016) with genotypes that are not only important in local farming practices, but also have a wide range of genetic potential for grain yield.

The first experiment—under greenhouse conditions—was a 17 × 2 completely randomized factorial design (soybean genotypes × Ni doses), with four replicates. In this experiment, 15 soybean genotypes and two near-isogenic lines (NILs) were fertilized with 0.0 mg kg^−1^ or 0.5 mg of Ni kg^−1^ (Table 1). Positive urease (*Eu3*) and urease activity-null (*eu3-a*, formerly *eu3-e1*) NILs only differ between each other in the integrity of the *UreG* gene, which codifies an accessory protein necessary to Ni incorporation into urease (Tezotto et al., 2016).

**Table 1:** Summary of characteristics for 15 soybean genotypes and two near-isogenic lines with urease-positive (*Eu3*) and urease activity-null (*eu3-a*).

**Genotype**	**Company^a^**	**Patent^b^**	**Transgenic event**	**Maturity^c^**	**Growth habit**	**Grain initial Ni concentration (mg kg^−1^)**
7379	GDM	31763	MON87701 x MON89788	7.4	Indeterminate	1.26
7200	NIDERA	28708	GTS-40-3-2	6.4	Indeterminate	1.47
6510	GDM	30256	GTS-40-3-2	6.5	Indeterminate	2.84
2728	MONSOY	28121	MON87701 x MON89788	7.2	Indeterminate	1.83
7849	BAYER	29661	MON87701 x MON89788	7.8	Indeterminate	1.32
3730	MONSOY	28124	MON87701 x MON89788	7.3	Indeterminate	1.90
2158	TMG	31291	MON87701 x MON89788	5.8	Indeterminate	2.53
797	MONSOY	31211	MON87701 x MON89788	7.9	Indeterminate	1.38
6215	TMG	33040	MON87701 x MON89788	6.4	Indeterminate	2.25
690	GENEZE	30151	GTS-40-3-2	6.9	Indeterminate	1.94
2737	COODETEC	28992	GTS-40-3-2	7.3	Indeterminate	2.33
8015	COODETEC	33191	MON87701 x MON89788	8.0	Determinate	1.50
791	BAYER	25931	GTS-40-3-2	7.9	Indeterminate	1.75
1378	SYNGENTA	31435	MON87701 x MON89788	8.0	Determinate	1.47
620	TMG	33097	MON87701 x MON89788	6.2	Indeterminate	1.64
*Eu3*^d^	–	–	*eu3-a/eu3-a* x *Eu3/Eu3*	–	Determinate	1.57
*eu3-a*^d^	–	–	*eu3-a/eu3-a* x *Eu3/Eu3*	–	Determinate	1.58

a *Maintainer of genotype*.

b *Details about patent register can be found at Brazil (2016)*.

c *Maturity groups defined by Alliprandini et al. (2009)*.

d *Isogenic lines described in Tezotto et al. (2016)*.

The second experiment—under field conditions—reproduced the treatments adopted in the greenhouse, with 15 × 2 factorial design (soybean genotypes × Ni doses), with four replicates. The NILs (*Eu3* and *eu3-a*) were not cultivated in the field experiment.

### Cultivation conditions

In the greenhouse experiment, soybean plants were cultivated in 4-L pots filled with soil collected from a native forest. This soil was classified as *Latossolo Vermelho Amarelo distrófico típico* (Embrapa Soils, 2013), corresponding in US Soil Taxonomy (Soil Survey Staff, 1999) to the Ustox Sub-Order of Oxisol. Before sowing, soil pH was adjusted to 6.0 with soil application of 1.75 g of calcium carbonate kg^−1^ and 0.75 g of magnesium carbonate kg^−1^ in each pot. Nickel treatments comprised a control—0.0 mg of Ni kg^−1^—and 0.5 mg of Ni kg^−1^ applied to the soil as nickel sulfate (NiSO_4_.6H_2_O). The other macro and micronutrients were supplied via soil (except N) at the following rates: 200 mg of P kg^−1^ (Ca[H_2_PO_4_]_2_), 75 mg of P kg^−1^ + 100 mg of K kg^−1^ (KH_2_PO_4_), 50 mg of S kg^−1^ (MgSO_4_.7H_2_O), 5.0 mg of Cl kg^−1^ (MnCl_2_.4H_2_O), 5.0 mg of Mn kg^−1^ (MnSO_4_.H_2_O), 3.0 mg of Zn kg^−1^ (ZnSO_4_.7H_2_O), 1.0 mg of B kg^−1^ (H_3_BO_3_), 1.0 mg of Cu kg^−1^ (CuSO_4_.5H_2_O), 0.5 mg of Mo kg^−1^ ([NH_4_]_6_Mo_7_O_24_.4H_2_O), and 0.1 mg of Co kg^−1^ (CoSO_4_.7H_2_O). Soybean plants obtained N through inoculation of seeds with N_2_-fixing bacteria (*Bradyrhizobium japonicum*, strain SEMIA 5079 and *Bradyrhizobium elkanii*, strain SEMIA 5019). Soil physical and chemical characteristics after soil fertilization and pH correction are listed on Table 2. Greenhouse temperatures were kept at 28 ± 5°C during the day and 20 ± 5°C at night, by means of an automatic computer-controlled system. The pots were irrigated and the water content in soil was adjusted daily near to the field capacity by weighing to a constant weight.

**Table 2:** Chemical characterization and particle size distribution before sowing of the native forest soil *Latossolo Vermelho Amarelo distrófico típico* (Oxisol) used in the greenhouse experiment and the cultivated soil *Latossolo Vermelho Amarelo eutrófico típico* (Oxisol) used in the field experiment.

**Properties**	**Units**	**Method/Extractant**	**Greenhouse**	**Field**
Sand	g kg^−1^	Hydrometer	740	656
Silt	g kg^−1^	Hydrometer	30	154
Clay	g kg^−1^	Hydrometer	230	190
SOM	g kg^−1^	Colorimetric	16.0	39.0
pH	-	Water	6.0	6.5
Al	cmol_c_ kg^−1^	Potassium chloride	0.0	0.0
Al + H	cmol_c_ kg^−1^	Calcium acetate, pH 7.0	0.7	2.3
N	g kg^−1^	Kjeldahl	1.3	2.2
P	mg kg^−1^	Mehlich-1	27.8	34.4
K	mg kg^−1^	Mehlich-1	47.0	170
Ca	cmol_c_ kg^−1^	Potassium chloride	2.7	5.2
Mg	cmol_c_ kg^−1^	Potassium chloride	1.7	2.1
S	mg kg^−1^	Dicalcium phosphate	18.1	7.5
B	mg kg^−1^	Hot water	0.5	1.3
Cu	mg kg^−1^	Mehlich-1	1.0	2.3
Fe	mg kg^−1^	Mehlich-1	30.6	17.4
Mn	mg kg^−1^	Mehlich-1	7.2	78.0
Zn	mg kg^−1^	Mehlich-1	2.4	9.3
Ni	mg kg^−1^	Mehlich-1	<0.2^a^-0.6^b^	0.4^a^-0.7^b^

a *After fertilization with 0.0 mg of Ni kg^−1^*.

b *After fertilization with 0.5 mg of Ni kg^−1^*.

*Soil classification according to Embrapa Soils (2013)*.

*SOM, soil organic matter*.

In the field experiment, soybean plants were cultivated in 15-m^2^ plots (6 lines of 6.25 m, equally spaced by 0.4 m) in the city of São Gabriel do Oeste, the largest soybean producer region in Brazil. This region's weather, according to the Köppen-Geiger classification, is described as tropical with mesothermal characteristics (Cwa), with an average temperature of 25°C during the day and 19°C during the night, and an average annual precipitation of 1,625 mm. The experimental site is located at an altitude of 665 m. The soil of this experimental site, classified as *Latossolo Vermelho Amarelo eutrófico típico* (Embrapa Soils, 2013), corresponds also to an Oxisol, according to the Soil Taxonomy (Soil Survey Staff, 1999), and has an agricultural cultivation history of annual species. Nickel fertilization was performed via soil at a rate of 1.0 kg of Ni ha^−1^ (equivalent to 0.5 mg of Ni kg^−1^) as nickel sulfate (NiSO_4_.6H_2_O). A control treatment, i.e., 0.0 kg of Ni ha^−1^, was used as well. Other macro and micronutrients were supplied as follows: (1) via soil (except N): 80 kg of P ha^−1^ (Ca[H_2_PO_4_]_2_.H_2_O), 130 kg of K ha^−1^ (KCl), 2.0 kg of Mn ha^−1^ (MnSO_4_.H_2_O), 4.0 kg of Zn ha^−1^ (ZnSO_4_.7H_2_O), 1.5 kg of B ha^−1^ (H_3_BO_3_), and 1.5 kg of Cu ha^−1^ (CoSO_4_.7H_2_O); and, (2) via seeds: 15 g of Mo ha^−1^ ([NH_4_]_6_Mo_7_O_24_.4H_2_O) and 2.0 g of Co ha^−1^ (CoSO_4_.7H_2_O). Soybean plants acquired N through inoculation of seeds with N_2_-fixing bacteria (*B. japonicum* and *B. elkanii*). Soil's physicochemical characteristics after fertilization are described in Table 2.

Expanded leaves in the flowering stage, i.e., the R1-R2 phenological stages, according to Fehr and Caviness (1977), were analyzed in both experiments for Ni and N concentration, for N metabolic compounds (urease, urea, ureides, and ammonia), as well as with regards to photosynthesis [SPAD index, electron transport rate (ETR), photochemical quenching (qP), non-photochemical quenching (qN), and maximum fluorescence (F_M_)].

Mature grains were harvested in the R8 stage (95% of the pods below 15% moisture, presenting mature pod color), for Ni and N concentration analysis and determination of grain yield.

For analyses in the greenhouse experiment, two plants per pot were collected, while five plants per plot were collected, pooled, and divided into uniform sub-samples for analyses in the field experiment.

### Grain yield evaluation

Soybean grains produced in each experiment were harvested and weighed for grain yield determination. In the greenhouse, yield estimate was done by collecting grains produced by each plant in the pot, divided by the number of plants, while in the field, grain yield was assessed by harvesting the two central lines of soybean in each plot. Grain yield was converted to dry weight by the correction of 13% moisture. The moisture was determined with an automatic measuring device (Gehaka G650i, Brazil).

### Nickel and nitrogen determination in leaf and grain

Nickel and N concentration in the leaves (the third leaf from the top of the plants) and the grains were determined in oven-dried (at 60°C, till constant weight) materials. For determination of Ni, 0.25 g of ground-dried plant material was digested in a closed-vessel microwave system (CEM Mars 5, US), using 30% hydrogen peroxide and 65% nitric acid. The final Ni concentration was determined through inductively coupled plasma-optical emission spectrometry (Perkin Elmer Optima 5300, US). Certified reference materials NIST® SRM® 1573a (tomato leaves) and BCR® 414 (plankton) were used for QA/QC protocols. Readings below 0.2 mg of Ni kg^−1^ were considered as not detectable and so not used for calculations. For determination of N, 0.35 g of ground-dried plant material were measured using elementary analyzer (Vario EL, German).

### Analysis of photosynthesis

Photosynthetic function was determined on the third leaf from the top of the plants. As previously mentioned, soybean plants photosynthesis was evaluated by measuring the SPAD index, as well as ETR, qP, qN, and F_M_. Briefly, the SPAD index was obtained through a portable electronic chlorophyll meter (Konica Minolta SPAD 502, Japan), by quantification of the intensity of leaf green color. To calculate the qP, qN, and ETR parameters (White and Critchley, 1999), *a*-chlorophyll fluorescence and light curve were determined. For the determination of *a*-chlorophyll fluorescence, intact leaves were measured between 8:00 a.m. and 12:00 noon, using a modulated pulse fluorometer (Heinz Walz Mini-PAM, Germany). To obtain the light curves, leaves were exposed to nine pulses of actinic (photosynthetic active) light, with increasing intensities (0–6,500 mol m^−2^ s^−1^) at intervals of 40 s, using the fluorometer. In order to obtain F_M_, leaves were kept in darkness for a minimum of 2 h to inactivate the photochemical phase. Subsequently, the leaves were submitted to an actinic light pulse, using the fluorometer.

### Evaluation of N metabolism

Urease activity and the major metabolic compounds involved in N metabolism (urea, ureides, and ammonia) were quantified in the fourth leaf collected from the top of the plants. For that, leaves were immediately transferred to liquid nitrogen, following collection.

For determination of leaf urease activity, a modified method described by Hogan et al. (1983), was used. Extraction was done with 8.0 mL of phosphate buffer at pH 7.4 for each 0.3 g of fresh material, which was incubated during 1 h at 30°C. One 0.5-mL aliquot was collected and added to 2.5 mL of reagent 1 (0.1 M phenol; 170 μM of sodium nitroprusside) and 2.5 mL of reagent 2 (0.125 M sodium hydroxide; 0.15 M dibasic sodium phosphate; sodium hypochlorite - 3% of Cl_2_). Samples were then incubated at 37°C for 35 min. Urease activity was determined by colorimetry (color intensity) in a spectrometer (Shimadzu UV-1280, Japan) at 625 nm absorbance.

Leaf urea concentration was measured through a modified procedure proposed by Kyllingsbæk (1975). Extraction was done with 1.0 mL of 10 mM formic acid for each 0.5 g of fresh material, under agitation. The extract was centrifuged at 13,200 RPM during 5 min, at 4°C. One 150-μL aliquot was collected and added to 3.0 mL of color developing reagent. Such reagent was prepared using a 1:1 proportion of the colorimetric reagent (7% [v/v] 0.2 M diacetylmonoxime; 7% [v/v] 0.05 M thiosemicarbazide) with the acid reagent (20% [v/v] sulphuric acid; 0.06% [v/v] 74 mM ferric chloride hexahydrate; 9% [v/v] ortho-phosphoric acid). Samples were incubated during 15 min at 99°C, under agitation, then kept in dark in an ice-cooled system for 5 min. Urea concentration was determined by colorimetry (color intensity) at 540 nm absorbance.

Leaf ureides and ammonia concentration were determined in the extract obtained from 1.0 g of fresh material in 10 mL of solution (60% [v/v] methanol; 25% [v/v] chloroform). The extract was centrifuged at 13,200 RPM during 5 min. Subsequently, the supernatant was collected to determine these compounds.

Total ureide concentration (allantoin and allantoic acid), as an indicator for BNF, was quantified through the methodology proposed by Vogels and Van der Drift (1970). One 300-μL extract aliquot was added to 500 μL of solution 1 (50% [v/v] 0.5 N sodium hydroxide; 50% [v/v] 0.15 N hydrochloric acid). The mixture was incubated at 100°C during 5 min. These solutions were then cooled to ambient temperature. Subsequently, the mixture was added to solution 2 (11.5% [v/v] 0.4 M phosphate buffer at pH 7; 11.5% [v/v] phenyl hydrazine; 70% [v/v] 0.65 N hydrochloric acid at −20°C; 7% [v/v] potassium ferrocyanide). Ureides concentration was determined through colorimetry (color intensity) at 535 nm absorbance.

Finally, ammonia concentration was quantified according to McCullough (1967). For that, one 150-μL extract aliquot was added to 2.0 mL of colorimetric solution. This solution was prepared using a 1:1 proportion of phenol reagent (2.5 g phenol and 12.5 mg sodium nitroprusside in 250 mL) with the phosphate reagent (1.25 g sodium hydroxide, 13.4 g monobasic sodium phosphate, and 2.5 mL 5% sodium hypochlorite in 250 mL). Samples were incubated at 37°C during 1 h. Ammonia concentration was then determined by colorimetry (color intensity) at 630 nm absorbance.

### Statistical analysis

Statistical analysis was performed through a two-way analysis of variance (ANOVA) and mean values were compared by the Dunnett's test (*P* = 0.05).

In order to assess the Ni treatment's overall effect on soybean N metabolism (leaf urea, ureides, and ammonia concentration, and urease activity), as well as on leaf N concentration and grain yield, a partial principal component analysis (PCA) was made for each experiment individually (greenhouse and field conditions). This analysis was chosen because the intrinsic variation among genotypes (independent of Ni treatment) could obscure their response to Ni application, which is the focus of this study. The marginal effect of genotypes was partialled out by subtracting each variable from its overall mean (irrespective to Ni treatment) for each genotype, prior to PCA analysis, resulting in a partial PCA (pPCA) as detailed in Legendre and Legendre (2013). This procedure does not change the interaction between genotypes and Ni treatments, but place all genotypes on a common scale, facilitating the visualization of how their responsiveness varies with Ni application.

## Results

Analysis of variance of the greenhouse experiment revealed that soybean plant response was dependent on genotypes and Ni doses (A x B) for leaf Ni concentration, grain Ni concentration, grain yield, urease activity, ammonia concentration, urea concentration, SPAD index, ETR, and qN (Table 3). For leaf N concentration, grain N concentration and ureides concentration, the effect of Ni fertilization was independent of the genotypes. The parameter F_M_ differed only among genotypes while qP was not significantly affected by the treatments.

**Table 3:** Two-way analysis of variance of 15 soybean genotypes and two near-isogenic lines (NILs) cultivated in greenhouse and field fertilized with 0.0 mg of Ni kg^−1^ and 0.5 mg of Ni kg^−1^.

**Source of variation – Greenhouse**					
	**Ni leaf**	**N leaf**	**Ni grain**	**N grain**	**Grain yield**
Genotype (A)	^**^	^**^	^**^	^**^	^**^
Ni dose (B)	^**^	^**^	^**^	^**^	^**^
A x B	^**^	n.s.	^**^	n.s.	^**^
CV (%)	6.2	7.2	15.7	5.1	3.2
	**Leaf ammonia**	**Leaf urea**	**Leaf urease**	**Leaf ureides**	**SPAD index**
Genotype (A)	^**^	^**^	^**^	^**^	^**^
Ni dose (B)	^**^	^**^	^**^	^**^	^**^
A x B	^**^	^**^	^**^	n.s.	^*^
CV (%)	14.7	26.7	12.9	18.3	5.5
	**ETR**	**qP**	**qN**	**F**_M_	
Genotype (A)	^**^	n.s.	^**^	^*^	
Ni dose (B)	^**^	n.s.	n.s.	n.s.	
A x B	^**^	n.s.	^*^	n.s.	
CV (%)	12.5	24.9	16.2	1.1	
**Source of variation – Field**					
	**Ni leaf**	**N leaf**	**Ni grain**	**N grain**	**Grain yield**
Genotype (A)	^**^	^**^	^**^	^**^	^**^
Ni dose (B)	^**^	^**^	^**^	^**^	^**^
A x B	^**^	n.s.	^**^	^*^	^*^
CV (%)	13.6	6.5	16.3	6.0	13.3
	**Leaf ammonia**	**Leaf urea**	**Leaf urease**	**Leaf ureides**	**SPAD index**
Genotype (A)	^**^	^**^	^**^	^**^	^**^
Ni dose (B)	^**^	^**^	^**^	^**^	^**^
A x B	^*^	^**^	^**^	^*^	n.s.
CV (%)	12.2	14.7	1.8	28.7	4.2
	**ETR**	**qP**	**qN**	**F**_M_	
Genotype (A)	^**^	^**^	^**^	^**^	
Ni dose (B)	^**^	n.s.	n.s.	n.s.	
A x B	n.s.	n.s.	n.s.	n.s.	
CV (%)	13.9	20.6	15.7	9.8	

*n.s., not significant by F-test*.

* *significant by F-test at P < 0.05*.

** *significant by F-test at P < 0.01*.

*Number of replicates, 4*.

*Degrees of freedom, greenhouse: A - 16; B - 1; A x B - 16; Residue - 102*.

*Degrees of freedom, field: A - 14; B - 1; A x B - 14; Residue - 87*.

*qP, photochemical quenching*.

*qN, non-photochemical quenching*.

*F_M_, maximum fluorescence*.

*ETR, electron transport rate*.

*CV, coefficient of variation*.

*The NILs were not tested in the field experiment*.

For the field experiment, ANOVA indicated, as observed in greenhouse experiment, a significant interaction between Ni fertilization and genotypes (A × B) for leaf Ni concentration, grain Ni concentration, grain N concentration, grain yield, urease activity, as well as ammonia, urea and ureides concentrations (Table 3). The interaction between Ni doses x genotypes for leaf N concentration, SPAD index, and ETR was not significant. The parameters qP, qN, and F_M_ differed only among genotypes.

Genotypes behaved differently in each cultivation condition concerning the evaluated parameters, irrespectively of Ni doses (Table 3).

Soil extractable Ni concentration after soybean cultivation increased with Ni fertilization by ~2.6 times in the greenhouse soil (from < 0.20 to 0.52 mg kg^−1^), and by ~1.7 times in the field soil (from 0.40 to 0.69 mg kg^−1^).

### Grain yield

**Figure 1:**
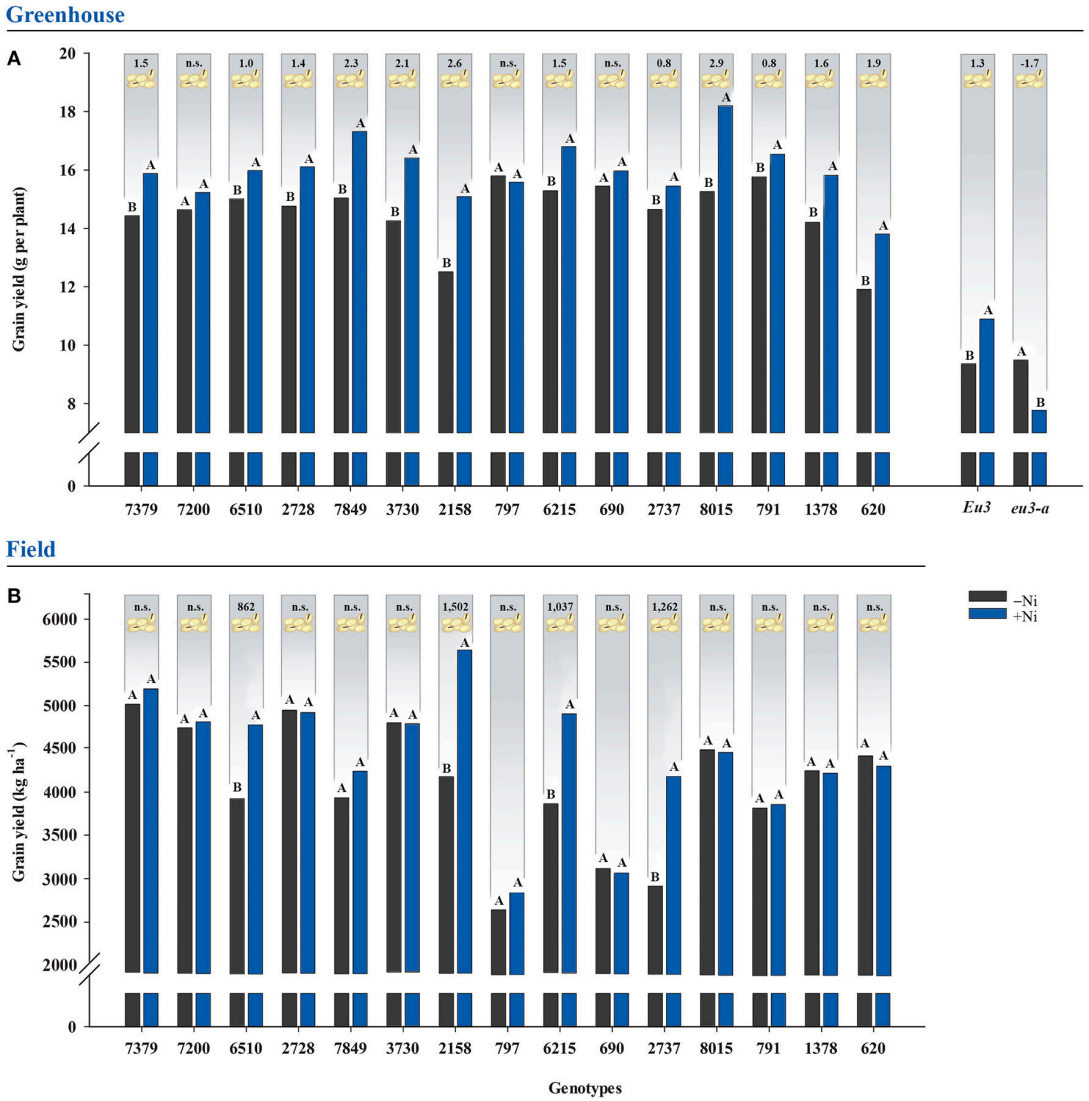
Effects on grain yield due to fertilization with 0.0 mg of Ni kg^−1^ (−Ni) and 0.5 mg of Ni kg^−1^ (+Ni) in 15 soybean genotypes and two near-isogenic lines (NILs, *Eu3* and *eu3-a*) cultivated in **(A)** greenhouse and **(B)** field conditions. Means were compared by the effect of the Ni doses in each genotype by Dunnett's test at *P* < 0.05, and those followed by the same letter do not differ. Values indicated in the upper part of the figure correspond to the amplitude of difference between Ni doses in grain yield. Grain yield was corrected to 13% of moisture. n.s., not significant. The NILs were not tested in the field experiment.

Nickel fertilization of greenhouse-grown soybean plants promoted increases in grain yield for 12 out of 15 genotypes evaluated and for the *Eu3* isogenic line, with increases of up to 2.9 g per plant (Figure 1). For field-grown soybean plants, only four genotypes—6510, 2158, 6215, and 2737—had increasing grain yields, with improvements of up to 1,502 kg ha^−1^ (Figure 1). The *eu3-a* mutant was the only treatment to express toxicity with Ni fertilization, as the addition of Ni reduced grain yield by 1.7 g per plant (Figure 1).

### Nickel and nitrogen concentration in aboveground plant tissues

Soil application of Ni resulted in higher leaf Ni concentration in all soybean genotypes in both cultivation conditions, i.e., greenhouse and field (Table 4). Nickel fertilization of soybean in the greenhouse promoted average increases in leaf Ni concentration of 1.5 times in genotypes (mean values without and with Ni ranged from 0.95 to 1.42 mg kg^−1^), and of 2.6 times on NILs (0.97 to 2.56 mg kg^−1^). The field-grown plants showed an average increase of 2.2 times in leaf Ni concentration (mean values without and with Ni ranged from 0.62 to 1.34 mg kg^−1^) (Table 4).

**Table 4:** Effects in leaf Ni and N concentration and grain Ni and N concentration due to fertilization with 0.0 mg of Ni kg^−1^ (−Ni) and 0.5 mg of Ni kg^−1^ (+Ni) in 15 soybean genotypes and two near-isogenic lines (NILs, *Eu3* and *eu3-a*) cultivated in greenhouse and field conditions.

**Genotype**	**Leaf Ni (mg kg**^**−1**^**)**		**Leaf N (g kg**^**−1**^**)**		**Grain Ni (mg kg**^**−1**^**)**		**Grain N (g kg**^**−1**^**)**	
	**−Ni**	**+Ni**	**−Ni**	**+Ni**	**−Ni**	**+Ni**	**−Ni**	**+Ni**
**GREENHOUSE**								
7379	1.18 B	1.51 A	35.7 B	40.7 A	2.45 A	1.70 B	55.1 B	62.2 A
7200	1.03 B	1.57 A	32.1 B	37.6 A	1.39 B	1.85 A	56.0 B	62.9 A
6510	0.83 B	1.57 A	40.7 B	45.9 A	1.38 B	1.89 A	56.7 B	65.5 A
2728	1.22 B	1.54 A	36.4 B	41.3 A	1.82 A	1.45 A	54.0 B	61.1 A
7849	1.00 B	1.22 A	34.3 B	39.5 A	1.48 A	1.55 A	56.5 B	61.3 A
3730	1.02 B	1.35 A	34.4 B	39.4 A	1.52 A	1.66 A	62.5 B	65.9 A
2158	0.99 B	1.90 A	36.6 B	41.5 A	1.86 B	2.68 A	56.3 B	59.1 A
797	1.27 B	1.65 A	37.8 B	42.8 A	1.45 A	1.47 A	61.6 B	62.9 A
6215	1.00 B	1.70 A	36.6 B	41.7 A	1.20 B	2.02 A	53.7 B	62.3 A
690	1.06 B	1.62 A	34.1 B	41.2 A	1.86 A	1.81 A	58.3 B	63.9 A
2737	0.75 B	1.17 A	33.3 B	39.3 A	1.36 A	1.67 A	63.6 B	67.8 A
8015	0.99 B	1.31 A	30.4 B	36.0 A	1.53 A	1.94 A	54.0 B	61.3 A
791	0.80 B	1.30 A	39.7 B	44.6 A	1.40 A	1.60 A	59.0 B	61.1 A
1378	0.76 B	1.01 A	30.3 B	35.4 A	1.56 A	1.88 A	60.0 B	61.3 A
620	0.40 B	0.88 A	26.5 B	32.5 A	1.82 B	2.52 A	54.0 B	56.9 A
*Eu3*	0.84 B	2.33 A	35.2 B	40.6 A	1.64 A	2.00 A	59.9 B	61.3 A
*eu3-a*	1.09 B	2.78 A	37.8 B	37.2 A	2.26 A	1.73 B	62.3 B	59.2 A
**FIELD**								
7379	0.45 B	1.57 A	53.5 B	54.9 A	1.22 B	2.66 A	54.2 B	61.5 A
7200	1.30 B	2.01 A	52.6 B	54.1 A	1.40 B	2.04 A	47.1 B	56.3 A
6510	0.81 B	1.28 A	57.8 B	60.2 A	2.29 B	3.07 A	53.6 A	53.7 A
2728	0.54 B	1.55 A	54.7 B	56.5 A	1.60 B	2.27 A	57.2 A	56.3 A
7849	0.85 B	1.79 A	50.7 B	52.5 A	1.30 A	1.65 A	58.0 A	57.6 A
3730	0.39 B	0.93 A	50.4 B	53.0 A	1.86 A	2.13 A	56.0 A	56.6 A
2158	0.31 B	0.65 A	59.5 B	61.3 A	1.91 A	2.20 A	56.7 A	57.6 A
797	0.35 B	0.92 A	42.3 B	44.9 A	1.39 B	1.89 A	59.1 A	58.2 A
6215	0.41 B	1.86 A	56.3 B	59.0 A	1.58 A	1.99 A	58.2 A	57.3 A
690	0.34 B	1.36 A	40.6 B	43.5 A	1.66 B	2.19 A	56.7 A	57.7 A
2737	1.51 B	2.26 A	55.7 B	57.6 A	1.59 B	2.34 A	58.8 A	58.8 A
8015	0.63 B	1.15 A	45.9 B	52.8 A	1.44 B	2.49 A	54.8 A	55.8 A
791	0.39 B	0.74 A	51.6 B	56.3 A	1.53 B	2.37 A	56.9 A	57.8 A
1378	0.56 B	1.01 A	51.8 B	53.8 A	1.34 B	2.20 A	54.2 B	60.1 A
620	0.51 B	0.97 A	50.9 B	55.6 A	1.75 A	1.71 A	52.0 B	57.6 A

*Means were compared by the effect of the Ni doses in each genotype by Dunnett's test at P < 0.05, and those followed by the same letter do not differ*.

*The NILs were not tested in the field experiment*.

Greenhouse-grown plants generally did not translocate more Ni to grains when fertilized with this micronutrient (Table 4). Among the 17 genotypes evaluated, 10 showed no increase in grain Ni concentration (mean values without and with Ni ranged from 1.56 to 1.70 mg kg^−1^), two of them—7379 and *eu3-a*—had a decrease (2.36 to 1.72 mg kg^−1^), and only five—7200, 6510, 2158, 6215, and 620—presented an increase in Ni concentration (1.53–2.19 mg kg^−1^). On the contrary, among the 15 field-grown soybean genotypes, 10 showed an increased in grain Ni concentration (mean values without and with Ni ranged from 1.55 to 2.35 mg kg^−1^) and five—7849, 3730, 2158, 6215, and 620—did not (1.68 to 1.94 mg kg^−1^).

Nitrogen in leaf and grain presented a behavior similar to that verified for Ni concentration in soybean aboveground tissues (Table 4). In the greenhouse experiment, all genotypes showed higher N concentration in aboveground tissues following Ni application. The average increase was by 1.1 times in soybean leaves (mean values without and with Ni ranged from 34.8 to 39.8 g N kg^−1^ with Ni), and of 1.1 times in grains (57.9 to 62.1 g N kg^−1^). Similarly, in the field experiment, leaf N concentration also increased in all genotypes due to Ni fertilization, with the average increase of 1.1 times (mean values without and with Ni ranged from 51.6 to 54.4 g N kg^−1^) (Table 4). However, this improvement on leaf N concentration did not result in higher grain N concentration, which occurred only in four—7379, 7200, 1378, and 620—out of the 15 genotypes (mean values without and with Ni ranged from 51.9 to 58.9 g N kg^−1^) (Table 4).

### Photosynthesis

**Figure 2:**
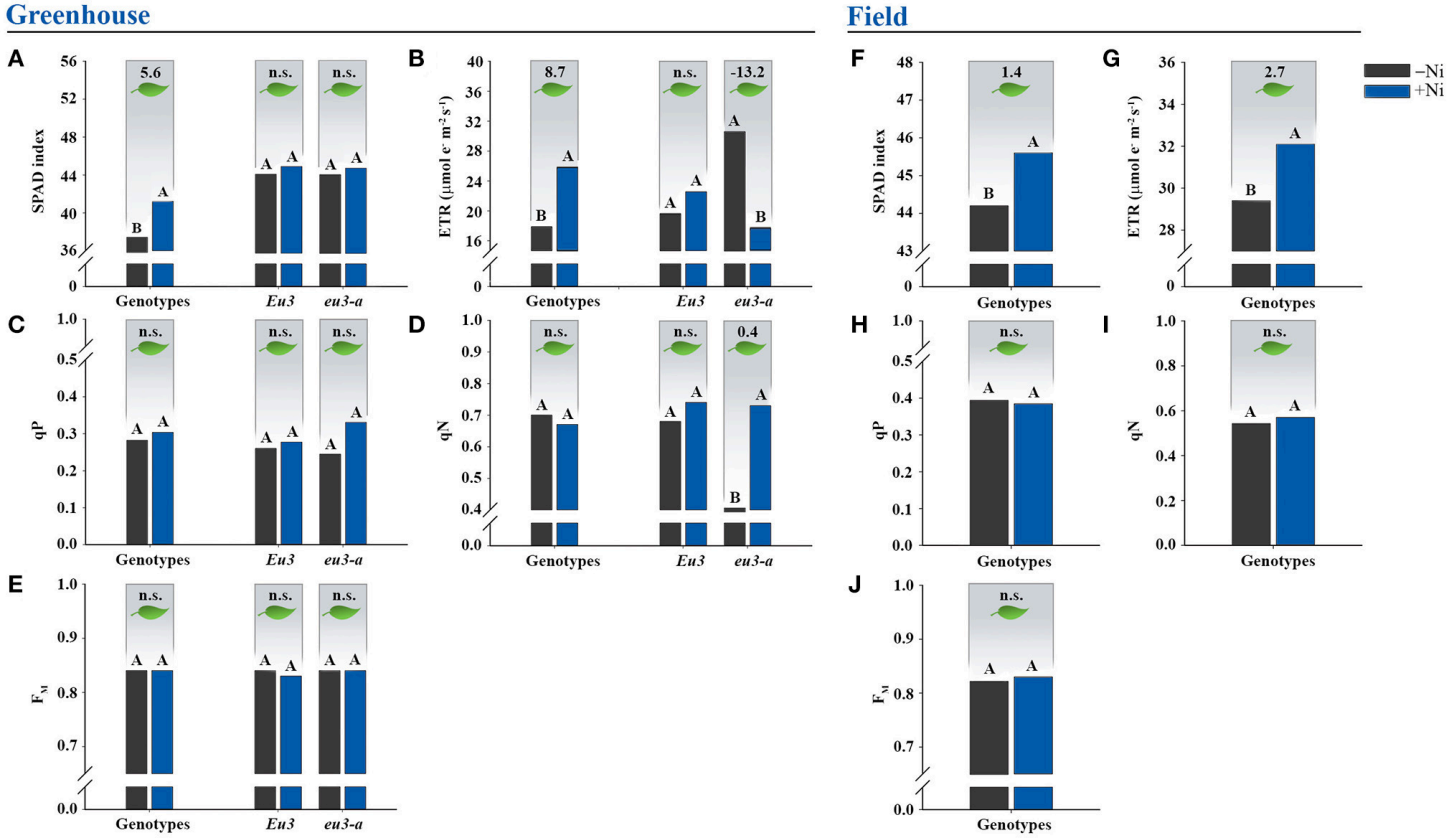
Effects on leaf photosynthesis due to fertilization with 0.0 mg of Ni kg^−1^ (−Ni) and 0.5 mg of Ni kg^−1^ (+Ni) in 15 soybean genotypes and two near-isogenic lines (NILs), *Eu3* and *eu3-a*, cultivated in **(A–E)** greenhouse condition and **(F–J)** field condition. Means were compared by the effect of the Ni doses in each genotype by Dunnett's test at *P* < 0.05, and those followed by the same letter do not differ. In greenhouse, only the mean of Ni-dose effects in the genotypes were presented since interaction genotype x Ni dose was caused by NILs alone. Values indicated in the upper part of the figure correspond to the amplitude of difference between Ni doses in photosynthesis. n.s., not significant. ETR, electron transport rate. qP, photochemical quenching. qN, non-photochemical quenching. F_M_, maximum fluorescence. The NILs were not tested in the field experiment.

Nickel fertilization in soybean genotypes affected positively the photosynthetic activity (Figure 2). For these variables, only the mean of Ni-dose effects in the genotypes were presented, since the interaction of genotype x Ni dose was caused by NILs alone (data not shown).

Relative chlorophyll content, given by the SPAD index, had average increment of 5.6 in the greenhouse experiment and of 1.4 in the field experiment with Ni application (Figures 2A,F). A higher efficiency of the photosystem II (PSII) was also verified by increases in ETR values in both conditions (greenhouse and field), with average increment of 8.7 μmol e^−1^ m^−2^ s^−1^ in the greenhouse-grown plants and 2.7 μmol e^−1^ m^−2^ s^−1^ in field-grown plants when Ni was applied (Figures 2B,G). The parameters qP, qN, and F_M_ were not affected by Ni fertilization (Figures 2C,D,E,H,I,J).

Concerning Ni fertilization in NILs, *Eu3* did not show response in the photosynthesis (Figures 2A–E). On the other hand, the Ni-fertilized *eu3-a* plants reduced ETR by 13.2 μmol e^−1^ m^−2^ s^−1^ (Figure 2B) and increased qN value by 0.4 (Figure 2D).

### N metabolism

#### Leaf urease activity

Leaf urease activity was very responsive to Ni fertilization (Table 5). Sixteen out of 17 soybean cultivars grown under greenhouse had higher activity of this enzyme when fertilized with Ni, except for the *eu3-a* mutant, which is unable to codify urease activation protein. Under field conditions, only five genotypes (7200, 2728, 690, 791, and 1378) did not show increases on the activity of this enzyme following Ni fertilization. Average increments of urease activity were up to 1.9 times in the greenhouse and 1.1 times in the field (Table 5).

**Table 5:** Effects on the leaf N metabolism due to fertilization with 0.0 mg of Ni kg^−1^ (−Ni) and 0.5 mg of Ni kg^−1^ (+Ni) in 15 soybean genotypes and two near-isogenic lines (NILs, *Eu3* and *eu3-a*) cultivated in greenhouse and field conditions.

**Genotype**	**Urease activity (**μ**mol g FW**^**−1**^ **h**^**−1**^**)**		**Ureides (**μ**mol g FW**^**−1**^**)**		**Ammonia (**μ**mol g FW**^**−1**^**)**		**Urea (**μ**mol g FW**^**−1**^**)**	
	**−Ni**	**+Ni**	**−Ni**	**+Ni**	**−Ni**	**+Ni**	**−Ni**	**+Ni**
**GREENHOUSE**								
7379	8.0 B	16.5 A	13.4 B	18.3 A	5.0 B	8.0 A	27.8 A	11.2 B
7200	8.6 B	16.5 A	16.1 B	18.7 A	4.7 B	9.4 A	19.2 A	14.4 A
6510	8.0 B	14.6 A	17.7 B	26.2 A	3.5 B	9.4 A	42.5 A	7.4 B
2728	9.7 B	15.1 A	18.1 B	23.5 A	7.5 B	10.5 A	25.2 A	23.0 A
7849	9.0 B	13.0 A	19.1 B	24.3 A	7.1 B	10.1 A	14.2 A	13.0 A
3730	8.2 B	19.4 A	16.7 B	21.8 A	6.1 B	9.1 A	44.2 A	31.2 B
2158	8.4 B	22.4 A	22.2 B	32.6 A	2.5 B	12.6 A	44.0 A	12.2 B
797	8.6 B	15.2 A	11.8 B	12.9 A	6.1 A	6.3 A	22.5 A	24.8 A
6215	9.9 B	17.3 A	11.7 B	20.4 A	1.3 B	5.0 A	26.7 A	12.3 B
690	9.2 B	16.2 A	17.0 B	17.7 A	5.6 A	6.2 A	12.7 A	10.3 A
2737	10.3 B	15.7 A	12.9 B	21.1 A	4.7 B	8.0 A	22.4 A	7.5 B
8015	10.5 B	16.0 A	15.2 B	20.8 A	8.9 B	11.6 A	15.9 A	8.4 A
791	8.1 B	15.4 A	14.6 B	19.8 A	7.6 B	11.5 A	34.2 A	17.8 B
1378	9.3 B	14.9 A	19.6 B	24.8 A	6.6 B	9.5 A	45.2 A	28.2 B
620	8.1 B	21.4 A	20.8 B	26.1 A	6.7 B	9.8 A	34.3 A	32.9 A
*Eu3*	9.3 B	20.5 A	20.0 B	30.1 A	11.3 B	14.3 A	45.8 A	10.0 B
*eu3-a*	6.8 A	6.9 A	15.1 B	14.6 A	10.0 A	10.0 A	85.8 B	98.2 A
**FIELD**								
7379	11.1 B	11.9 A	26.4 A	33.3 A	11.5 B	15.1 A	37.5 A	15.2 B
7200	13.5 A	13.5 A	26.4 A	33.6 A	11.9 B	15.1 A	25.9 A	19.5 A
6510	13.4 B	14.1 A	22.8 B	37.1 A	12.6 B	18.7 A	32.7 A	5.7 B
2728	11.1 A	11.2 A	18.8 A	25.2 A	12.9 B	16.3 A	34.0 A	31.0 A
7849	10.6 B	11.3 A	21.5 A	29.6 A	9.5 B	13.7 A	19.1 A	17.6 A
3730	11.0 B	11.7 A	18.3 A	19.7 A	11.6 B	15.1 A	59.7 A	42.1 B
2158	10.5 B	13.8 A	16.3 B	41.6 A	12.3 B	18.7 A	33.9 A	9.4 B
797	11.0 B	11.6 A	26.7 A	26.9 A	12.2 A	11.8 A	30.4 A	33.5 A
6215	12.7 B	14.2 A	22.6 B	36.2 A	13.2 B	19.0 A	20.6 A	9.4 B
690	11.8 A	11.9 A	25.7 A	27.0 A	13.3 A	13.4 A	17.1 A	14.0 A
2737	12.6 B	12.9 A	24.2 B	35.0 A	12.2 B	17.5 A	17.2 A	5.8 B
8015	11.9 B	12.7 A	11.8 A	13.2 A	11.1 B	14.9 A	21.5 A	18.1 A
791	11.5 A	11.8 A	15.7 A	18.5 A	11.0 B	14.2 A	46.2 A	24.1 B
1378	10.8 A	11.1 A	17.0 A	21.3 A	9.7 B	13.6 A	61.0 A	38.0 B
620	10.6 B	13.0 A	17.0 A	17.0 A	10.1 B	13.7 A	46.3 A	44.5 A

*Means were compared by the effect of the Ni doses in each genotype by Dunnett's test at P < 0.05, and those followed by the same letter do not differ*.

*The NILs were not tested in the field experiment*.

*FW, fresh weight*.

#### Leaf ureide concentration

Nickel fertilization positively affected the synthesis of total ureides (allantoin and allantoic acid), which are the main way of exporting N fixed by nodules to other soybean plant tissues (Table 5). Nickel fertilization in the greenhouse-grown soybean promoted increases in ureide concentration for all 17 genotypes, with an average increment of 1.3 times. For field-grown soybean, only four (6510, 2158, 6215, and 2737) out of the 15 genotypes had higher ureide concentration in response to Ni fertilization, with average increments of 1.8 times in leaf ureide concentration (Table 5).

#### Leaf ammonia concentration

As ammonia is a product from urea hydrolysis, its leaf concentration was also very responsive to Ni fertilization, indicating, thus, that this micronutrient improved N assimilation in plants (Table 5). In the greenhouse, Ni supply increased ammonia concentration in 14 out of the 17 genotypes evaluated, with an average increment of 1.9 times. Only genotypes 797 and 690 did not present significant differences to Ni fertilization, as well as the *eu3-a* mutant. Under field conditions, exactly the same genotypes responded to Ni fertilization, with an average increase in ammonia concentration of 1.4 times (Table 5).

#### Leaf urea concentration

A higher urease activity due to Ni fertilization is expected to reduce leaf urea concentration. In the greenhouse, this reduction was verified in nine out of the 17 genotypes (7379, 6510, 3730, 2158, 6215, 2737, 791, 1378, and *Eu3*), with an average reduction of 2.9 times (Table 5). In contrast, the *eu3-a* mutant presented an increase of 1.1 times in urea concentration. Under field-grown conditions, exactly the same genotypes presented reduction in leaf urea concentration in response to Ni fertilization, with an average reduction of 2.7 times (Table 5).

**Figure 3:**
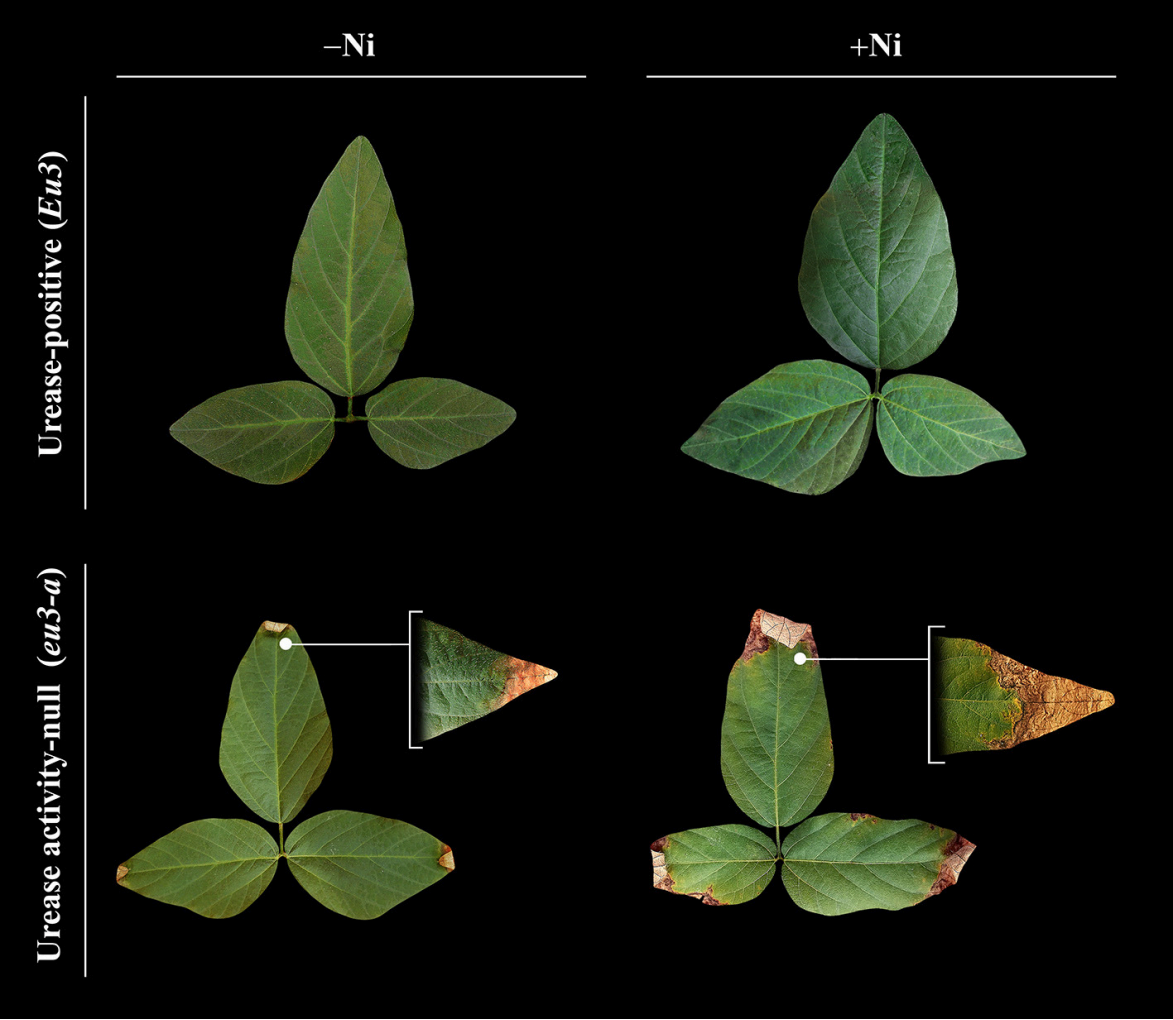
Contrast of leaves of two near-isogenic soybean lines at flowering stage, urease-positive (*Eu3*) and urease activity-null (*eu3-a*), fertilized with 0.0 mg of Ni kg^−1^ (−Ni) and 0.5 mg of Ni kg^−1^ (+Ni). Independently of Ni dose, *Eu3* line developed normally while *eu3-a* line presented symptoms of hyponasty and initial necrosis lesions on leaflet tips. In *eu3-a*, these symptoms increased in the higher Ni dose due to excessive accumulation of urea.

Regarding NILs, the *eu3-a* mutant, even without Ni fertilization, always presented the highest leaf urea concentration, with an average of 85.8 μmol g FW^−1^, a value that was 1.9 times higher than that verified for *Eu3* (Table 5). When Ni fertilized, *eu3-a* showed an expressive accumulation of urea—98.2 μmol g FW^−1^—while *Eu3* was able to hydrolyze this molecule, resulting in only 10.0 μmol g FW^−1^ of urea. In addition, the excessive urea accumulation in *eu3-a* leaves caused visible lesions in the leaflet tips (Figure 3). Such lesions contained a very high level of urea, with an average concentration of 576 μmol g FW^−1^.

#### Principal components analysis (PCA)

**Figure 4:**
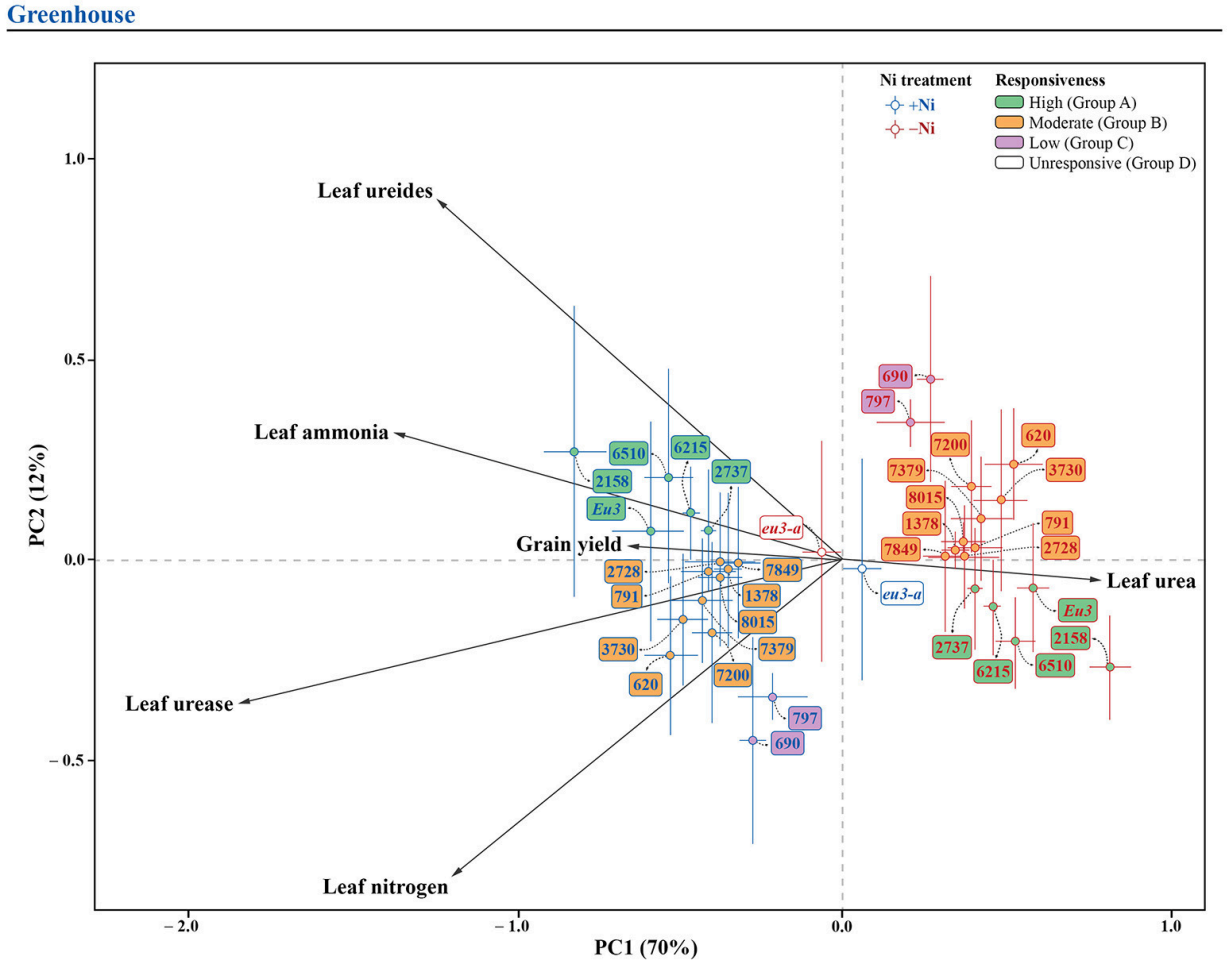
Biplot of partial principal components analysis of the variables related to N metabolism, leaf N concentration and grain yield for 15 soybean genotypes and two near-isogenic lines (NILs, *Eu3* and *eu3-a)*, fertilized with 0.0 mg of Ni kg^−1^ (−Ni) and 0.5 mg of Ni kg^−1^ (+Ni), cultivated in greenhouse condition. In the figure, genotypes are divided into four groups according to responsiveness of N metabolism to Ni fertilization: Group A, high; B, moderate; C, low; and D, unresponsive.

**Figure 5:**
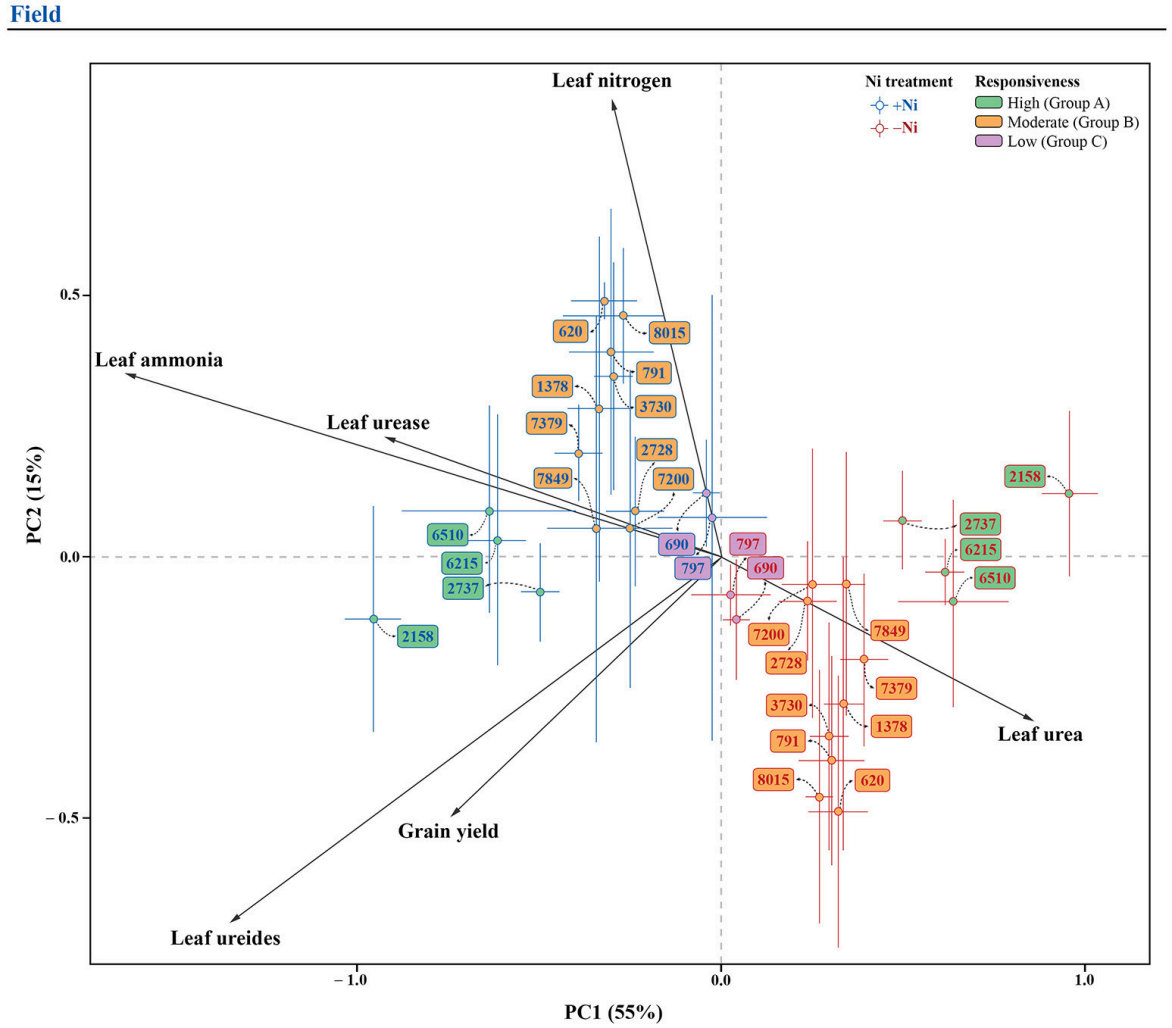
Biplot of the partial principal components analysis of variables related to N metabolism, leaf N concentration and grain yield for 15 soybean genotypes, fertilized with 0.0 mg of Ni kg^−1^ (−Ni) and 0.5 mg of Ni kg^−1^ (+Ni), cultivated in field condition. In the figure, genotypes are divided into three groups according to responsiveness of N metabolism to Ni fertilization: Group A, high; B, moderate; and C, low.

In order to promote a better understanding of the overall Ni fertilization effect on soybean yield, leaf N concentration, leaf ammonia, leaf ureides, leaf urea, and urease activity for each genotype, two pPCA were performed (one for each experiment), with the marginal effect of genotype (overall mean for each genotype, independently of Ni treatment) being partialled out. For the greenhouse experiment, the first two principal components represented 82% of total variation (Figure 4), whereas, for the field experiment, the first two components represented 70% of total variation (Figure 5). In both experiments, the first component (horizontal axis) represented most of the total variation and clearly separated treatments with and without Ni fertilization. Grouping of the samples receiving Ni toward the left side of the pPCA biplot indicates increased grain yield, leaf N concentration, leaf ammonia, leaf ureides, and urease activity, associated with decreases in leaf urea, with the opposite for mutant *eu3-a* (Figures 4, 5).

Soybean genotypes were separated in groups by their responsiveness to Ni fertilization, as follows: (1) In the greenhouse experiment: Group A (high response)—6510, 2158, 6215, 2737, and *Eu3*; Group B (moderate response)—7379, 7200, 2728, 7849, 3730, 8015, 791, 1378, and 620; Group C (low response)—797 and 690; Group D (unresponsive—*eu3-a* (Figure 4); and, (2) In the field experiment: the same groups were evident, except for NILs, which were not studied under this condition (Figure 5).

## Discussion

In both greenhouse and field conditions, initial soil Ni concentration (~0.3 mg kg^−1^) and initial grain Ni concentration (~1.8 mg kg^−1^) were not high enough to supply soybean plant-specific requirements (Tables 1, 2), since Ni fertilization via soil led to physiologic enhancements (Figure 2), better N metabolism (Table 5), and higher grain yield (Figure 1). The lack of Ni-deficiency symptoms associated with these results revealed a hidden Ni deficiency. To the best of our knowledge, this is the first study to report a hidden deficiency of this micronutrient in soybean under field conditions. Previous studies, although being carried out on greenhouse-grown soybean plants alone, corroborate the Ni performance verified in this study. Kutman et al. (2013) and Lavres et al. (2016) demonstrated that Ni fertilization induces yield gains, while Kutman et al. (2014) showed that soybean seeds with Ni concentration up to 5.35 mg kg^−1^ did not express their maximum yield and, thus, an external supply of this micronutrient was required. Thus, these previous results give support to our data, indicating a higher grain yield in soybean plants when fertilized with Ni. Our study also revealed that not all soybean genotypes respond in the same way to Ni fertilization, since despite improvements in the photosynthetic apparatus (Figures 2A,B,F,G) and a better N metabolism (Table 5), when supplied with Ni, some of the soybean genotypes did not produce higher grain yield (Figure 1).

Based on our data, the genotypes were separated into groups of Ni responsiveness based on the responses of their N metabolism (Table 5 and Figures 1, 4, 5).

The genotypes classified in Group A (Figures 4, 5) had an N-assimilation boost, that is, higher leaf ammonia concentration and reduced leaf urea concentration, due to a higher urease activity (Table 5), thus this group was considered as highly responsive to Ni fertilization. To be able to transport N-urea to N-sink tissues, soybean plants produce ammonia, as result of urea hydrolysis by urease activity (Wang et al., 2008; Witte, 2011; Polacco et al., 2013; Winter et al., 2015). According to Mokhele et al. (2012) and Ohyama et al. (2017), when degraded, ammonia produces different amino acids, so that a higher free amino acid pool affect positively several plant-growth process, especially secondary compounds synthesis. Although little is known about Ni influences ammonia metabolism in plants, Bai et al. (2006, 2007) observed that pecan plants under low Ni supply showed an inhibition in the shikimate pathway, disrupting the conversion of free amino acids to other products and, thus, blocking some N pathways. Moreover, the genotypes in this group also had the higher increases in ureides synthesis, products of purine degradation and main form of N transport from nodules, during BNF, to aboveground parts in legume plants (Rentsch et al., 2007). As observed by Lavres et al. (2016), yield increases in soybean plants due to Ni fertilization resulted from a more efficient BNF, probably due to a higher activity of hydrogenase. In addition, Todd and Polacco (2004), studying soybean, confirmed that urea and ammonia might be direct products of ureides degradation in urease pathway. Regardless of the cultivation condition, i.e., greenhouse or field, the genotypes in Group A had increases in grain production.

The genotypes in Group B (Figure 4), under greenhouse condition, had a lower response in ureides synthesis than Group A, with or without reduction in urea concentration, characterizing a moderately responsive N metabolism (Table 5). In this case, usually a higher yield was found due to Ni supply (Figure 1). Field-conditions were more restrictive since the genotypes in this group presented no yield increases (Figures 1, 5), associated mainly with no increases in ureides (Table 5). Thus, our data revealed that the absence of response to Ni fertilization in any step of N metabolism might result in lack of yield gains, in which some compounds are more limiting than others. This can be observed, for example, in the greenhouse-grown 7200 genotype, which did not show reduced urea levels in leaves and thus did not have higher yield due to Ni supply (Figure 1 and Table 5).

The genotypes in Group C (Figures 4, 5), showed low response in N metabolism when Ni fertilized in both conditions. In this group, soybean plants lacked response in leaf ammonia, with this N compound being the key factor that limits productivity gains (Figure 1 and Table 5).

Group D (Figure 4), with no response in N metabolism to Ni supply, comprised the *eu3-a*—urease activity-null. This mutant has a blockage in ammonia synthesis, via urease, and thus, had a significant accumulation of leaf urea with Ni fertilization, which caused toxicity symptoms (Figure 3 and Table 5). The excessive urea accumulation resulted in lower grain yield (Figure 1). This emphasizes the critical role of Ni in N metabolism.

A more efficient N metabolism with Ni supply is corroborated by the higher N concentration in the leaves (Tables 4, 5). According to Kutman et al. (2013), soybean plants increased N concentration by up to 30% when fertilized with Ni, indicating that this micronutrient improves internal N utilization efficiency and N remobilization.

With Ni supply, we verified a higher Ni concentration in soybean leaves, as was observed for N concentration. However, higher concentrations of Ni and N in the leaf were not always related to a higher grain concentration (Table 4). Thus, our data indicate that the translocation rate for these nutrients is controlled by phenotype-specific properties. According to Belimov et al. (2016), the phenotypic specificity can modulate homeostasis and regulation of transporters for many ions. Moreover, since Ni absorption by roots of soybean can be via passive diffusion or active transport (Seregin and Kozhevnikova, 2006; Yusuf et al., 2011), the relative Ni concentration may vary among genotypes. The same phenotype-specific effect on grain yield, photosynthesis, and N metabolism indicated that the cultivation conditions influenced genotypes response to Ni fertilization (Figures 1, 2 and Tables 3, 5).

Since many farmers all over the world have used Ni fertilization without clear evidence of its need for crop growth, there are concerns about a possible toxicity of this element in cultivated plants (Kretsinger et al., 2013). Our data revealed that a soil-applied Ni rate of 0.5 mg kg^−1^ resulted in Ni leaf concentrations up to 2.26 mg kg^−1^ and Ni grain concentrations up to 3.07 mg kg^−1^ (Table 4). These values are well below the levels considered toxic to plants, which are > 10 mg kg^−1^ in sensitive species, > 50 mg kg^−1^ in moderately tolerant species, and > 1,000 mg kg^−1^ in Ni hyperaccumulator plants (Seregin and Kozhevnikova, 2006; Chen et al., 2009; Yusuf et al., 2011).

Some photosynthetic parameters considered as stress indicators also confirmed the absence of Ni toxicity in the soybean genotypes. The quenchings, qP and qN, are protective mechanisms that plants employ to dissipate energy from photochemical processes and should only be accessed by plants in case of light stress (Ashraf and Harris, 2013; Dall'Osto et al., 2017). Therefore, the lack of responses of qP and qN with Ni fertilization indicates that plants did not experience oxidative damage in PSII reaction centers (Figures 2C,D,H,I). Moreover, according to Baker (2008), healthy leaves have F_M_ values of ~0.8, which is similar to the value found in the genotypes, even when Ni fertilized (Figures 2E,J). Positive photosynthetic responses, ETR and SPAD index, increased in Ni-fertilized plants (Figures 2A,B,F,G), indicating a more efficient photosynthetic apparatus in the soybean genotypes.

The *eu3-a* mutant accumulated toxic levels of urea in leaves, even without Ni supply (Table 5). With addition of 0.5 mg of Ni kg^−1^ via soil, urea toxicity symptoms were intensified, being also associated with Ni-toxicity symptoms (Figure 3). The toxic level of Ni (Table 4) was high enough to reduce the mutant's growth (data not show) and ETR (Figure 2B), and increase the stress indicator qN (Figure 2D). Aiming to obtain the Ni-toxicity symptoms in soybean plants, Reis et al. (2017) observed formation of brown color on leaves induced by the presence of Ni inside cells, similarly to what was observed in the *eu3-a*.

Finally, concerning food safety of Ni fertilization in soybean plants, we first need to set the maximum allowable daily intake (ADI) of Ni for humans, which is expected to be 1.33 mg of Ni per day for an adult and 0.31 mg of Ni per day for a child. Such ADIs are based on a reference dose (RfD) for Ni of 0.02 mg of Ni kg^−1^ per day (Integrated Risk Information System, 1991), which was calculated from a no-observed-adverse-effect level (NOAEL) of 5.0 mg of Ni kg^−1^ per day (Ambrose et al., 1976; Institute of Medicine US and Panel on Institute of Medicine US Panel on Micronutrients, 2002), and a body mass of 66.6 kg for an adult and 15.4 kg for a child (Cole et al., 2007; Guilherme et al., 2015).

Next, assuming that a grain containing ~3 mg of Ni kg^−1^ in dry weight—the highest concentration of Ni in grains in this study—is used for assessing the risk of Ni ingestion via food chain, then a child needs to ingest >100 g of soybean grains (dry weight) per day in order to overcome a risk coefficient of 1. Such daily consumption of soybean is far beyond the recommended ingestion standards of *in natura* grains and soybean products. According to Do et al. (2007), the daily intake of *in natura* soybean grains is 2.5 ± 4.9 g (*n* = *708*). In Asian countries—the largest consumers of soybean—the daily intake of soybean and soy-related foods is 23.0 ± 18.2 g (Toyomura and Kono, 2002; Do et al., 2007; Katsuyama et al., 2009). Thus, the amount of Ni in soybean grains found in this study is considered safe and does not pose a threat to human health if direct consumption of grain is taking into account.

## Conclusions

Fertilization with a 0.5 mg of Ni kg^−1^ dose via soil resulted in higher grain yield in 12 greenhouse-grown genotypes and 4 field-grown genotypes, revealing a hidden Ni deficiency under both cultivation conditions. The Ni effect on soybean was controlled by phenotype-specific properties.

Yield increases resulted from a more efficient N metabolism, especially ureides. The higher ureides synthesis, possibly originated from a higher N_2_-fixation, and their catalysis by urease activity must result in higher ammonia concentration, so that increases in grain yield can be realized. The genotypes were separated into groups of Ni responsiveness based on the responses of their N metabolism: high response (with enhanced N metabolism), moderate response (limited by low ureides synthesis and/or urea synthesis), low response (limited by ammonia synthesis), and unresponsive (limited by urease activity).

Nickel fertilization resulted also in photosynthetic enhancements in soybean plants—especially in the photochemical phase—except for the *eu3-a*. Absence of ureolytic activity in this mutant resulted in a higher concentration of urea, which accumulated mainly in leaflet tips, resulting in a lower grain yield.

Thus, Ni fertilization at the dose employed in this study is beneficial for soybean and possibly for other annual species, in soils with low extractable-Ni, resulting in agronomical gains while meeting food safety standards. However, more studies are required to set an accurate Ni rate and to verify residual effects of Ni in the soil, especially for oxidic conditions prevalent in tropical agroecosystems. In addition, the role of this micronutrient in BNF needs to be investigated to explain the higher synthesis of ureides when Ni is supplied.

## Author contributions

DS and BW were in-charge for development of hypothesis, experiment conduction, data analysis, and writing of this manuscript. AR and FdB are experts in plant physiology, contributing mainly in the field experiment, and in review of this manuscript. TS is expert in statistical analysis and soil microbiology, contributing mainly in data analysis and in review of this manuscript. JS is expert in BNF, contributing in the understanding of how nickel affect N2-fixation process and in review of this manuscript. LG and MC are co-advisors and the coordinators of our research group. Their contributions extends to all steps of the research that led to this manuscript.

### Conflict of interest statement

The authors declare that the research was conducted in the absence of any commercial or financial relationships that could be construed as a potential conflict of interest.
